# Supplementary material for: Cooperative stator assembly of bacterial flagellar motor mediated by rotation
Source: Nat Commun. 2021 May 28;12:3218. doi: 10.1038/s41467-021-23516-y (PMC8163892; doi:10.1038/s41467-021-23516-y)
Supplement: Supplementary file 1 — Supplementary Information [file 41467_2021_23516_MOESM1_ESM.pdf]

Supporting Information for "Cooperative stator assembly of bacterial flagellar motor mediated by rotation"

Kenta I. Ito[a], **Shuichi Nakamura**[a], **and Shoichi Toyabe**[a, *]

[a]Department of Applied Physics, Graduate School of Engineering, Tohoku University, Aoba 6-6-05, Sendai 980-8579, Japan
[*]toyabe@tohoku.ac.jp

Supplmentary Figures

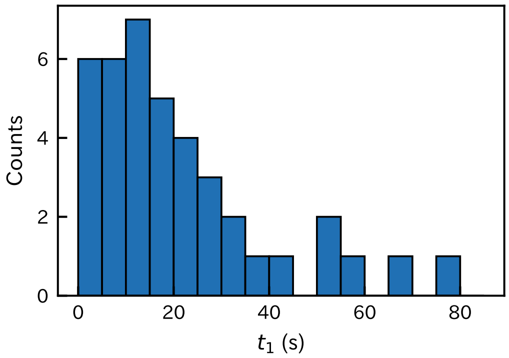

Supplementary Figure 1. The distribution of t_1, the timing of the stable stator-unit binding. The bin width is 5 s. The number of samples is 40. Source data are provided as a Source Data file.

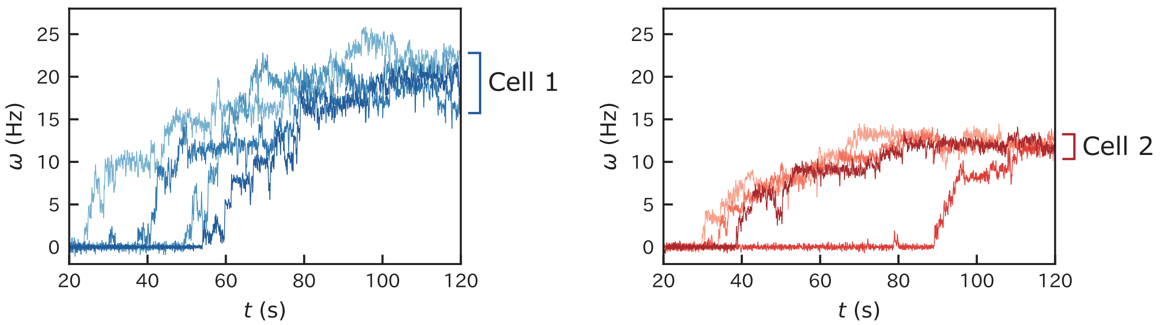

Supplementary Figure 2. The traces in Fig. 2b are separately plotted for comparison. Source data are provided as a Source Data file.

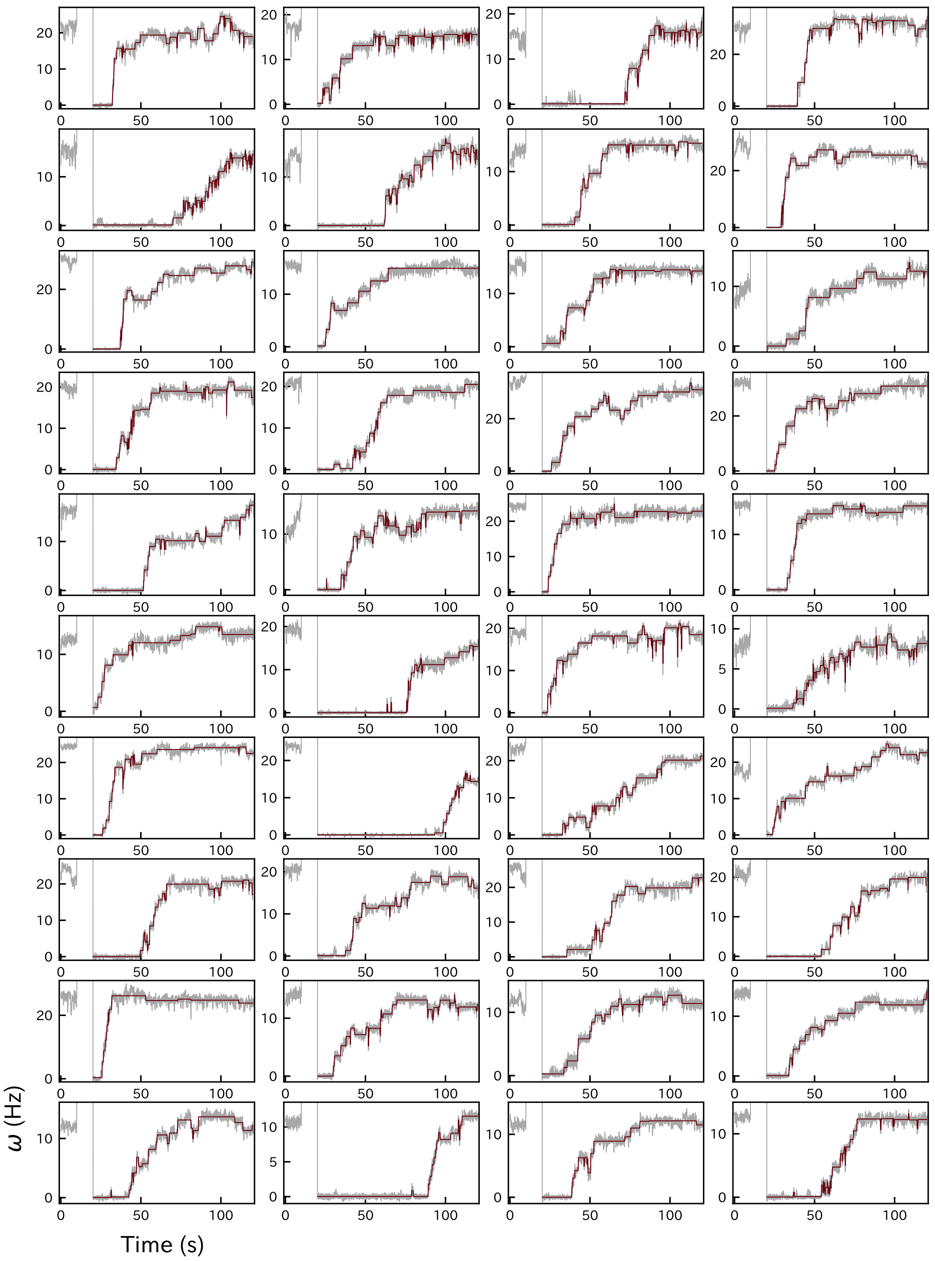

Supplementary Figure 3. The traces used for Fig. 2. The rotation traces smoothed by the moving average with the window length of 500 points and frame shift of 100 points (gray) are superposed by the step-analysis traces (red). Source data are provided as a Source Data file.

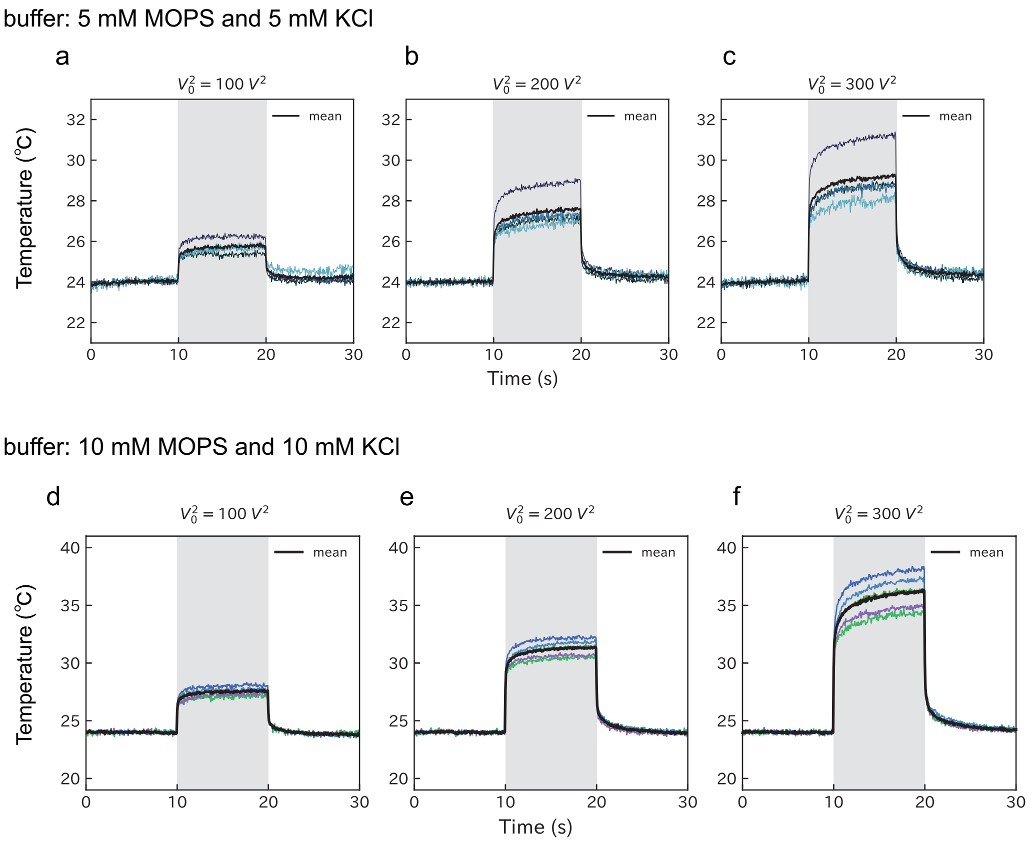

Supplementary Figure 4. Temperature rise by the induction of the electrorotation in the observation buffer containing 5 mM MOPS and 5 mM KCl (**a**–**c**) or 10 mM MOPS and 10 mM KCl (**d**–**f**). The electrorotation with the indicated magnitude was applied from 10 s to 20 s. Different colors correspond to the data measured in different independent chambers. The number of samples are four (**a**–**c**) or five (**d**–**f**). Black thick line indicates the mean. Source data are provided as a Source Data file.

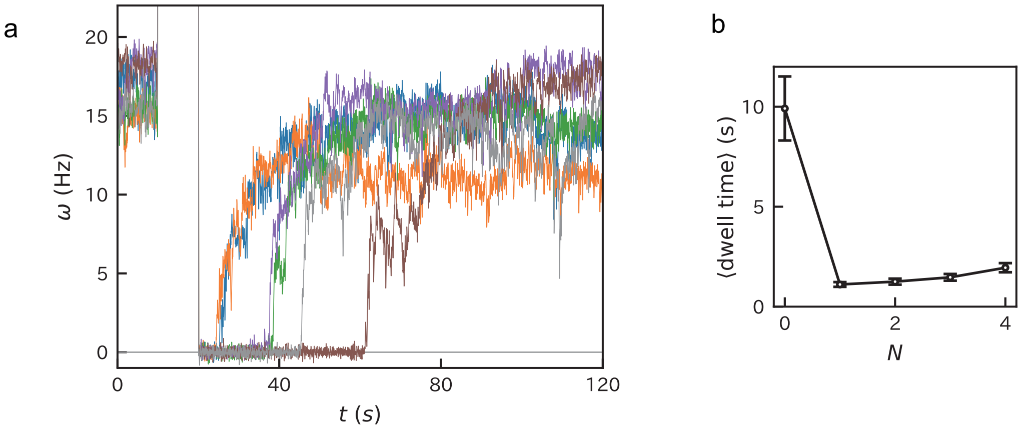

Supplementary Figure 5. Typical remodeling traces (**a**) and dwell time (**b**) without a shearing treatment, blocking agent with reduced concentration (50 mg/ml Perfect Block instead of 250 mg/ml), and observation buffer containing high ion strength (10 mM MOPS and 10 mM KCl). Different colors correspond to different runs of a same cell. Data in **b** are presented as the mean values ± standard errors. The number of traces used for the analysis is 38. The number of dwells are 75, 111, 122, 152, and 184 for $N = 0, \ldots, 4$, respectively. Source data are provided as a Source Data file.

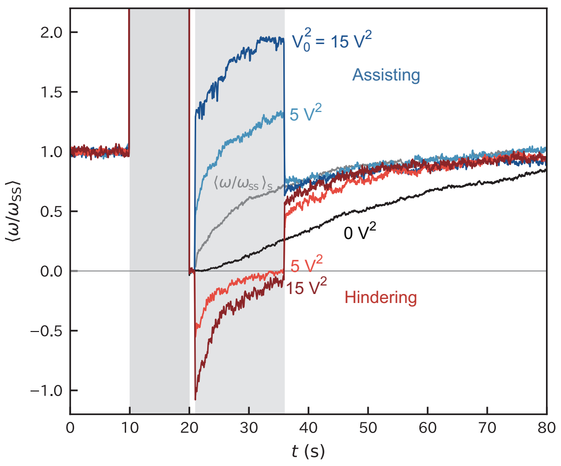

Supplementary Figure 6. The plots of Fig. 3a and b were combined for comparison.

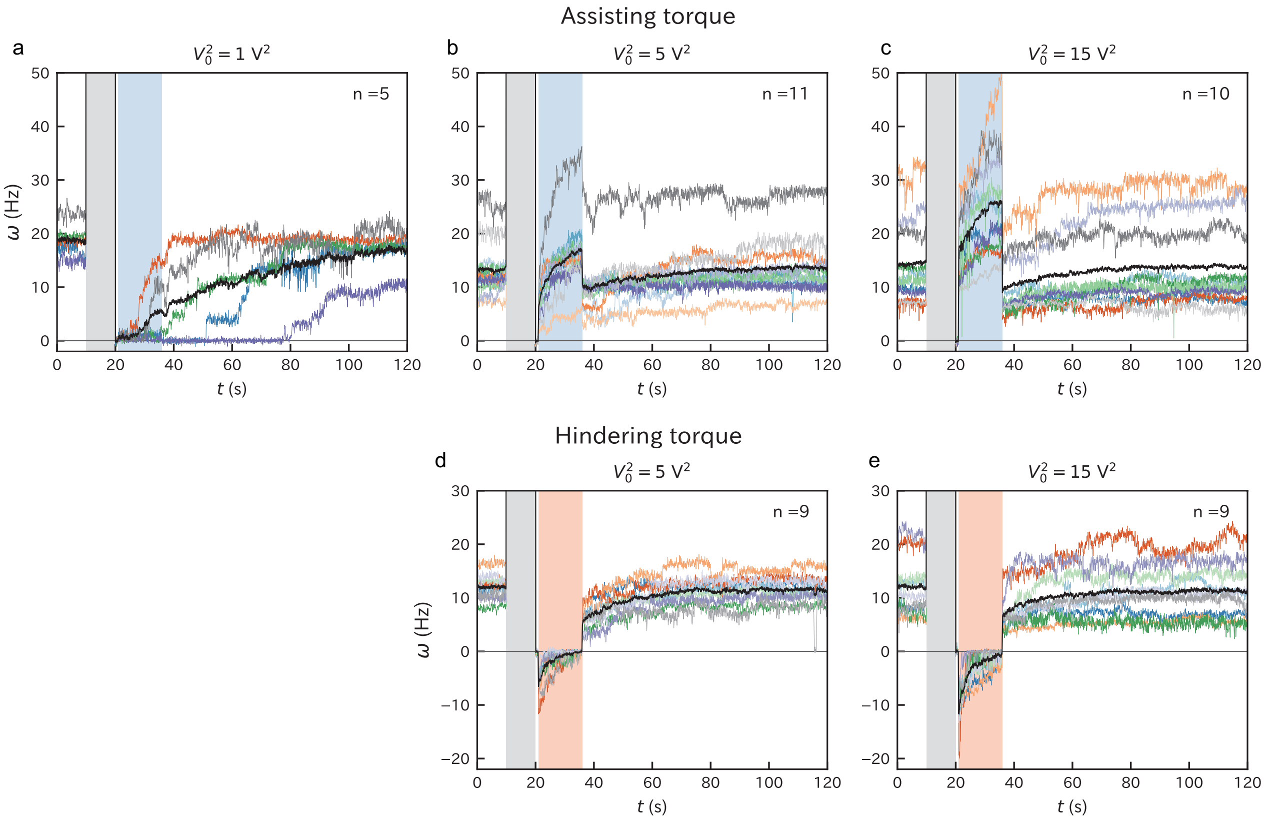

Supplementary Figure 7. Remodeling traces under forced rotation with different torque magnitude. Different colors correspond to different runs. Traces of multiple cells are mixed. Black curves correspond to the ensemble averages. Source data are provided as a Source Data file.

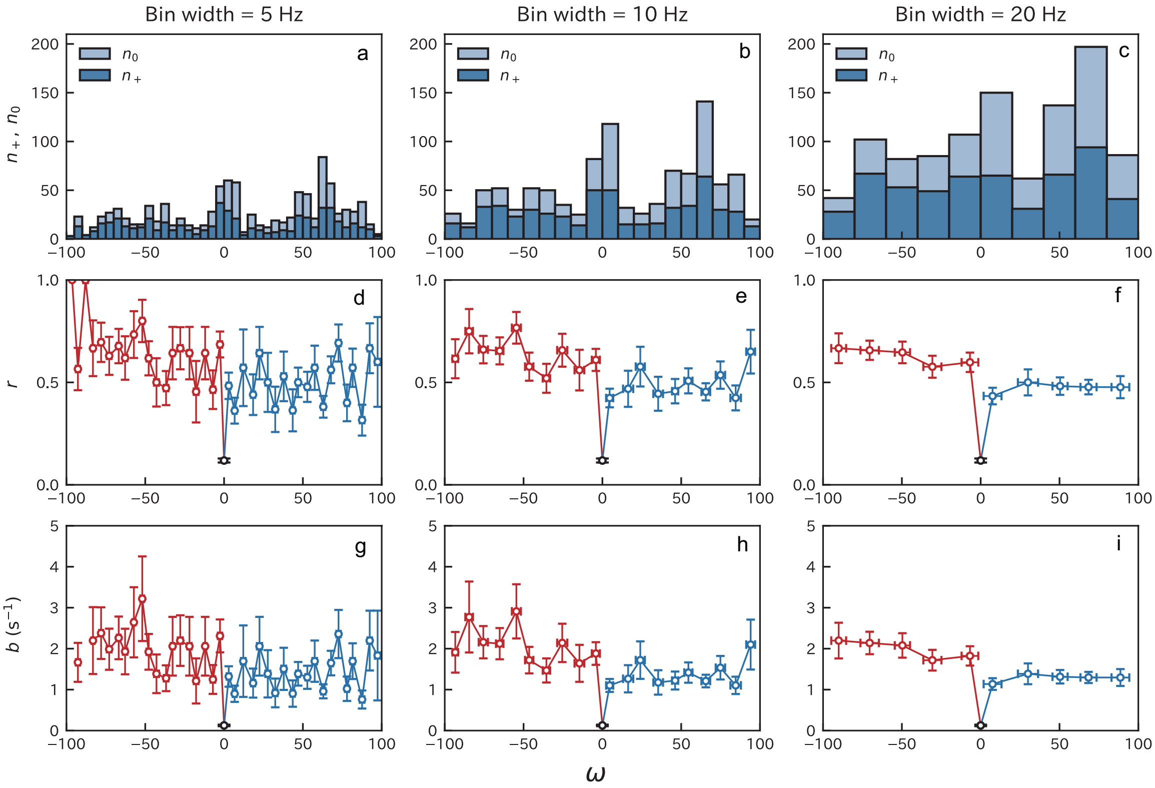

Supplementary Figure 8. Summary of the binding parameters with different bin widths. **a**–**c**, n_+ (blue) and n_0 (light blue). **d**–**f**, $r = n_+/(n_+ + n_0)$. Red and blue correspond to the CW and CCW rotations, respectively. **g**–**i**, the effective binding rate given by $b = -\tau^{-1}\ln(1-r)$. Data are presented as mean values $\pm$ standard errors. The number of samples correspond to $n_+ + n_0$ (**a**–**c**). Source data are provided as a Source Data file.

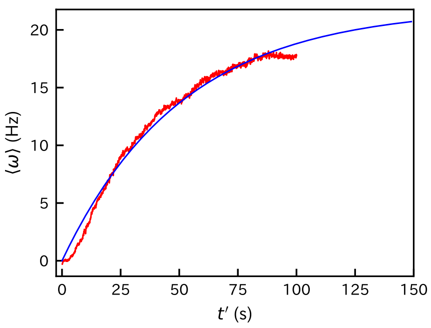

Supplementary Figure 9. Comparison of the averaged trace without electrorotation (red, $\langle\omega\rangle$ of Fig. 2c) with the fitting curve $v_{\max}(1 - e^{-t/\tau})$ (blue) obtained in Wadhwa et al. (Fig. S4 of ref.[1], 300 Hz). We used the same time constant $\tau = 51.1$ s. Since they used the number of the stator units as the vertical axis, we fitted the above curve and obtained $v_{\max} = 21.9$ Hz.

Supplementary Methods

1. Temperature rise

The temperature under electrorotation was measured using two fluorescent dyes, Rhodamine B (Sigma-Aldrich) and Rhodamine 101 (Sigma-Aldrich). These dyes have fluorescence with different dependence on the temperature. The spatial temperature gradient may induce a thermophoretic migration of the dyes. However, the ratio of the two dyes provides the temperature assuming that the thermophoretic magnitude is the same for these two dyes. The calibration curve was measured using a realtime thermal cycler (BioRad). The electrorotation chamber was first filled with 50 µg/ml Rhodamine B, 50 µg/ml Rhodamine 101, or observation buffer without fluorescent dye were flowed into the chamber. The fluorescence of these three solutions with/without electrorotation were measured at 16 Hz and analyzed to estimate the temperature profile (Supplementary Fig. 4).

The temperature rise by electrorotation in the observation buffer containing 5 mM MOPS and 5 mM KCl, which was the standard one int this work, was estimated to be 1.8 °C for $V_0^2 = 100\,\mathrm{V}^2$, 3.5 °C for 200 V^2, and 5.1 °C for 300 V^2 (Supplementary Fig. 4a, b, c). The temperature relaxed to the room temperature soon within a few seconds after the electrorotation was turned off. The typical value of V_0^2 during the assisting phase was 240 V^2, which yields the temperature increase of 4.1 °C by interpolating the above values.

On the other hand, in a high-ion-strength buffer containing 10 mM MOPS and 10 mM KCl used for the test (Supplementary Fig. 5), the temperature raised approximately 12 °C for 300 V^2 (Supplementary Fig. 4f), which was the typical voltage in Supplementary Fig. 5.

Supplementary References

1. Wadhwa, N., Phillips, R. & Berg, H. C. Torque-dependent remodeling of the bacterial flagellar motor. *PNAS* 201904577 (2019).
